# Dietary *verbena officinalis* reduces feather-pecking duration in laying ducks by modulating cecal microbiota composition and hypothalamic neurotransmitter secretion

**DOI:** 10.3389/fmicb.2025.1605305

**Published:** 2025-06-13

**Authors:** Ai Liu, Yongcai Zhu, Shenglin Yang, Bingnong Yao, Fuyou Liao, Baoguo Zhu

**Affiliations:** ^1^Key Laboratory of Animal Genetics, Breeding and Reproduction in the Plateau Mountainous Region, Ministry of Education, Guizhou University, Guiyang, China; ^2^College of Animal Science, Guizhou University, Guiyang, China; ^3^School of Materials Science and Engineering, Guizhou Minzu University, Guiyang, China

**Keywords:** cecal microbiota, duck, feather-pecking behavior, hypothalamic noradrenaline, *verbena officinalis*

## Abstract

*Verbena officinalis*, a traditional Chinese herb with antioxidant and anti-inflammatory properties, has not been extensively studied for its effects on poultry gut microbiota and behavior. This study evaluated the impact of *verbena officinalis* supplementation (0, 2, and 4%) on growth performance, cecal microbiota, and feather-pecking behavior in laying ducks. Ninety healthy 1-day-old ducks were randomly assigned to three dietary treatments for a 5-week growth trial, and 45 feather-pecking ducks were used to assess the behavioral and neurochemical effects. Before the trials, ducks were adapted to the experimental conditions for 7 days and fed a basal diet. The results showed no significant differences in body weight or average daily gain (ADG) among the groups (*p* > 0.05), but the feed conversion ratio (FCR) was significantly lower in *verbena* groups (*p* < 0.05). Cecal microbiota analysis revealed higher Chao 1 and ACE indices (*p* < 0.01), increased *Bacteroidetes* (*p* < 0.05), and decreased *Firmicutes* and *Megamonas* in the verbena groups (*p* < 0.05). *Spirochaetae* (*p* = 0.014), *Elusimicrobia* (*p* = 0.032), and *Lentisphaera* (*p* = 0.036) were the predominant differential phyla, whereas *Oscillospira* (*p* = 0.003), *Phascolarctobacterium* (*p* = 0.039), and *Megamonas* (*p* < 0.001) were the predominant differential genera. Feather-pecking duration and hypothalamic norepinephrine (NE) levels were reduced in verbena groups (*p* < 0.05), with NE negatively correlated with *Spirochaetae* (*p* < 0.05). In conclusion, 2% *verbena officinalis* supplementation promotes beneficial gut microbiota changes and reduces feather-pecking behavior, although the underlying mechanisms require further investigation.

## 1 Introduction

Feather pecking (FP) is a common pathological behavior in poultry (Cronin and Glatz, 2020) that results in plume and skin damage in recipients, causing tissue injury and pain (Savory, 1995). Severe FP can lead to cannibalism and eventually death (Rodenburg et al., 2013), which can have a negative impact on poultry production and welfare, as well as on economic aspects. The prevalence statistics, pathogenesis, nutritional regulation, and environmental improvement of pecking behavior in the majority of cases have been reported in laying hens, Pekin ducks, parrots, and geese (Jenkins, 2001; Dong et al., 2021; Wang et al., 2025). However, few studies have been conducted on laying ducks.

The causes of feather pecking include feeding conditions, genetic factors, stress, nutritional levels, and even light conditions (Lambton et al., 2010; Pan et al., 2025). Research on the pathogenesis of FP has focused on genomic, gut microbiota, neurology, and neurotransmitter characteristics associated with behavior, including psychogenic traits (Hierden et al., 2004; Kops et al., 2017; de Haas and van der Eijk, 2018). These studies considered FP behavior to be more internally regulated than an external factor. More studies have shown that FP phenotypes in laying hens are associated with differences in bidirectional communication in the microbiome-gut-brain axis (Mindus et al., 2021; Huang et al., 2023). Kraimi et al. suggested that FP is associated with the microbiota-gut-brain axis, which is an interaction between the gut microbiota, neurotransmitters, and the immune system (Kraimi et al., 2019). The communication of the gut-microbiota-brain axis is bidirectional. Gut microbiota-derived metabolites, hormones, and neurotransmitters not only modulate gut function but also communicate with the brain through vagal afferents (Cao et al., 2021). The hypothalamic–pituitary–adrenal (HPA) axis is activated by various stress stimuli, which may alter the gut microbiota composition and function.

Compared with low-pecking birds, high-pecking birds exhibit reduced peripheral serotonin (5-hydroxytryptamine, 5-HT) levels, altered gut microbiota composition, and impaired immune competence (Kops et al., 2017). Cecal l-lactate, d-lactate, total lactate, and short-chain fatty acids (SCFAs) were higher in high FP hens, and putrescine and cadaverine concentrations were higher in the ileum with low FP (Meyer et al., 2013). There were differences between laying hens with different FP phenotypes in peripheral and central metabolites and gut microbiota (Wang et al., 2023). Another study indicated that *L. rhamnosus* JB-1 could stimulate cecal velocity and amplitude of contractions, which were positively correlated with the feather-pecking number (van Staaveren et al., 2020).

Based on the study of the microbiota-gut-brain axis, researchers are currently exploring practical approaches to mitigate FP in poultry. Recent evidence suggests that the gut microbiome influences acute stress responses in ducks via gut-hypothalamic communication (Gu et al., 2025). Moreover, the glutamatergic system regulates FP behavior in laying hens by altering plasma arginine and histidine levels through the gut–brain axis (Yan et al., 2025). Tryptophan supplementation may reduce feather-pecking behavior in laying hens (Linh et al., 2021; Van Hieu et al., 2021). Supplementation with *Lactobacillus rhamnosus* prevents FP by increasing the immunological effect, maintaining a stable cecal microbiota, and affecting catecholamine concentrations in stress-induced feather pecking in chickens (Hierden et al., 2004), potentially reducing feather pecking in laying hens. In mule ducks, the consumption of a mannan-oligosaccharide (MOS) and *β*-glucan (BG) combination (MOS-BG prebiotics) at high doses (3 g/kg) may reduce feather-pecking behavior by decreasing dopamine and increasing serotonin levels in the plasma, whereas low doses (1.5 g/kg) have the opposite effect (Mahmoud et al., 2021). The effects of traditional Chinese medicine on feather-pecking behavior have been less extensively researched.

It is permissible to include traditional Chinese medicine (TCM) in feed to enhance livestock growth (Tian et al., 2021), and it is environmentally and livestock-friendly without the risk of drug resistance. Interactions between absorbable active small molecules of TCM and the gut microbiota can induce physiological changes (An et al., 2019). A study conducted in mice revealed that geissoschizine methyl (GM) ameliorated the isolation-induced increase in aggressiveness (Nishi et al., 2012). Shoukary et al. reported that adding 2% black seed (*Nigella sativa*) to the diet significantly decreased aggressive behavior and feed conversion ratio in adult pigeons (El Shoukary et al., 2018). Studies on the effects of TCM on ducks have focused on growth performance, egg and meat quality, antimicrobial effects, antioxidant activities, immune function, disease treatment, and intestinal health (Ao and Kim, 2020; Yang S. L. et al., 2020; Yao B. N. et al., 2023), but rarely on the gut microbiota or neurotransmitters associated with behavior.

*Verbena officinalis* is a traditional herbal medicine that is widely used in China and Europe (Khan et al., 2016; Polumackanycz et al., 2022). Verbena contains iridoids, phenylpropanoid glycosides, flavonoids, phenolic acids, terpenoids, and essential oils. Additionally, the main compounds were verbenalin (max. 6,196 mg/100 g DW), verbascoside (max. 2,264 mg/100 g DW), and hastatoside (max. 582 mg/100 g DW) (Kubica et al., 2020). Verbena has been studied for its antioxidant, antibacterial, analgesic, anti-inflammatory, anticonvulsant, and anticancer properties in humans. For animals, verbena has shown activity as an antioxidant and an immune stimulant in rainbow trout (Hoseinifar et al., 2020) and Avelignese horses (Palazzo et al., 2019), improved growth performance in piglets (Pastorelli et al., 2012), and possessed anticonvulsant, anxiolytic, and sedative activities in mice (Kubica et al., 2020). We are aware of only a few studies that have evaluated the effects of verbena on growth performance and gut microbiota in laying ducks.

Therefore, this study was designed to investigate the effects of common verbena powder on growth performance, gut microbiota, and hypothalamic neurotransmitter levels in laying ducks, with the aim of exploring its potential to mitigate feather-pecking behavior in this species.

## 2 Materials and methods

### 2.1 Animal ethics statement

This study was approved by the Subcommittee of Experimental Animal Ethics of Guizhou University, Guiyang, China (No. EAE-GZU-2022-E032), and ARRIVE guidelines.

### 2.2 Verbena powder

Verbena powder was purchased from Juchuntang Chinese Medicinal Materials Sales Co., Ltd., Bozhou City, Anhui Province. Verbena powder was obtained from dried common verbena (*verbena officinalis* L. or verbenaceae) leaves, stems, and roots. On a dry matter basis, verbena powder contains about 5.9 g/ 100 g crude protein, 70 g/ 100 g crude fiber, 1.5 g/ 100 g fat, 5.8 g /100 g ash, and 17 g/ 100 g available carbohydrates, and energy is about 244 kcal / 100 g (Content is provided by the sales company).

### 2.3 Animal, diet, and experiment design

Ducks were purchased from Xingluzhou Farm, Sansui County, Guizhou Province, and housed at the farm of Guizhou University. Three basal diets (Table 1) were formulated to meet or exceed the Sell et al. (1994) requirements for ducks, and the dietary treatments were (1) T1 (CON) control: basal diet without adding verbena; (2) T2 (2% V): basal diet with 2% verbena (20 g verbena/kg diet); (3) T3 (4% V): basal diet with 4% verbena (40 g verbena/kg diet). In the first growth experiment, a total of 90 healthy Sansui ducklings at 1 day old with an average initial body weight of 40.22 ± 0.26 g were randomly divided into 3 treatments with 3 replicates per treatment and 10 ducks per replicate. Ducks were placed in galvanized wire cages (0.98 m^2^) with 15 ducks per cage. All the cages were equipped with feeders, nipple drinkers, and raised plastic floors. Before the feeding trial, ducks were adapted to the experimental conditions for 7 days, and during this phase, they were fed with a basal diet. The growth experiment lasted for 5 weeks. For the second cecal microbiota experiment, when the ducks in the first experiment were fed at 11 weeks of age, the cecal contents of the ducks were collected to detect the microbial composition. For the feather-pecking behavior experiment, behavioral observations, cecal microbiota analysis, and hypothalamic neurotransmitter detection were conducted. A total of 45 Sansui feather-pecking ducks at 42 days of age with an average feather-pecking frequency of more than 10 times per hour were selected and randomly divided into three treatments, with 15 ducks per treatment. The ducks were placed in cages with 15 ducks per cage. The dosage was selected based on our previous studies of other Chinese herbal medicines in ducks (Yang S. L. et al., 2020; Yao B. N. et al., 2023).

**Table 1:** Ingredients and chemical composition of diets with and without *verbena officinalis*.

Items	**Starter (0–5 weeks)**			**Grower (6–12 weeks)**		
Ingredients, g/kg	T1(CON)	T2(2%V)	T3(4%V)	T1(CON)	T2(2%V)	T3(4%V)
Corn	63.85	62.85	61.85	64.85	63.85	62.85
Soybean meal	27.83	26.83	25.83	25.83	24.83	23.83
Wheat bran	1.50	1.50	1.50	1.50	1.50	1.50
Rapeseed cake	4.00	4.00	4.00	4.00	4.00	4.00
CaHPO_4_	1.50	1.50	1.50	2.50	2.50	2.50
Limestone	0.85	0.85	0.85	0.85	0.85	0.85
NaCl	0.25	0.25	0.25	0.25	0.25	0.25
Premix^1^	0.22	0.22	0.22	0.22	0.22	0.22
verbena powder	0.0	2.0	4.0	0.0	2.0	4.0
Analytical composition^2^						
Crude protein, g/kg	19.31	19.00	18.75	18.52	18.24	17.95
Metabolizable energy, MJ/kg	11.44	11.34	11.25	11.16	11.32	11.23
Calcium, g/kg	0.96	0.96	0.96	1.26	1.25	1.25
Available phosphorus, g/kg	0.43	0.43	0.42	0.66	0.65	0.65
Lysine, g/kg	1.04	1.00	0.98	0.99	0.96	0.93
Methionine, g/kg	0.29	0.28	0.27	0.28	0.28	0.27

^1^Premix provided (per kg): Cu:10 mg; Fe: 80 mg; Mn: 60 mg; Zn: 60 mg; I: 0.4 mg; Se: 0.2 mg; VA: 4000 IU; VD3: 1000 IU; VB2: 8 mg; VE: 20 IU; VK: 32 mg; VB1: 3.5 mg; Niacin: 50 mg; Folic acid: 1.0 mg; Calcium pantothenate: 10 mg; Pyridoxol: 2.5 mg; VB12: 0.01 mg; Biotin: 0.1 mg; Chloride choline: 500 mg. ^2^Calculated values.

In the first week, the temperature was 33°C, which was reduced by 1°C every week from the second to the fourth weeks. And from the fifth week to the twelfth week, the temperature was maintained at 25°C–28°C until the end of the experiment. During the experiment, all ducks were provided with sufficient clean water, the relative humidity was maintained at approximately 65–70%, and the houses were cleaned once a day.

### 2.4 Growth performance

During the growth period (day 42), all ducks were weighed once a week, and feed intake was recorded daily in the morning to calculate the average daily feed intake (ADFI). The average daily gain (ADG) and feed conversion rate (FCR) were calculated after the experiments were completed.

### 2.5 Sample collection

On day 42 of the growth experiment, all ducks in the three treatment groups were randomly selected. After 12 h of fasting, blood samples (3 mL) were collected from the wing vein and centrifuged at 3000 × g at 4°C for 10 min. When ducks were fed to 11-week-olds, 9 ducks from each treatment (3 per replicate) were randomly selected and sacrificed to collect the cecal contents under sterile conditions. The slaughter trial involved euthanasia using an overdose of pentobarbital sodium (100 mg/kg). This was done following the AVMA “Guidelines on Euthanasia of Animals: 2020 Edition,” “Laboratory animal—Guidelines for euthanasia” (GThisT 39,760–2021), and “Technical specifications on euthanasia of laboratory animals” (RB/T 061–2021) of the People’s Republic of China. Digesta samples of the cecum were promptly collected on ice and stored at −80°C for analysis of 16S RNA.

### 2.6 Serum biochemical parameters and immune index

Serum concentrations of total protein (TP), albumin (ALB), calcium (Ca), phosphorus (P), glucose, uric acid (UA), and urea (UREA) were measured using an automated biochemical analyzer (Mindray BS-240VET). Immunoglobulin G (IgG), immunoglobulin A (IgA), and immunoglobulin M (IgM) levels were analyzed using ELISA kits (Jiancheng Biotechnology Institute, Nanjing, China) (Yang S. L. et al., 2020).

### 2.7 Microbiota analysis

For cecal microbiota diversity, 3 ducks per replicate (n = 9 per treatment) were randomly selected and sacrificed to collect the cecal contents under sterile conditions at 11 weeks. The ducks were slaughtered after 12 h of fasting, and the slaughter trial animals were euthanized with an overdose of pentobarbital sodium (100 mg/kg). It was done following the AVMA “Guidelines on Euthanasia of Animals: 2020 Edition,” “Laboratory animal—Guidelines for euthanasia” (GB/T 39760–2021), and “Technical specifications on euthanasia of laboratory animals” (RB/T 061–2021) of the People’s Republic of China. The euthanasia method described above was used for this study. The QIAamp DNA Stool Mini Kit (Qiagen, Hilden, Germany) was used to extract total bacterial DNA from fecal samples, according to the manufacturer’s instructions. To analyze the abundance and diversity of the microbiota, 16S rRNA V3-V4 regions were sequenced on the Illumina NovaSeq 6,000 PE250 platform. Sequencing was performed using an Illumina NovaSeq 2 × 250 bp paired-end configuration (TinyGene Biotechnology Co. Ltd., Shanghai, China). Due to contamination of the final sample group during the detection process, the corresponding data were excluded from the analysis. As a result, the analysis was conducted on eight samples per treatment.

### 2.8 Feather-pecking behavioral observation

Media Record 2.0 and The Observer XI software were used to record and analyze the daily behavior of ducks. We observed 7 days after adding verbena. Feather pecking was defined as continuous pecking in the same duck or self-pecking for > 4 s (Birkl et al., 2017).

### 2.9 Feather pecking, cecal microbiota detection, and neurotransmitter analysis

At the end of the feather-pecking behavioral experiment, four ducks per treatment were randomly selected and slaughtered to collect cecal contents and hypothalamic tissues under aseptic conditions. Hypothalamic tissues were used for neurotransmitter detection using high-performance liquid chromatography-mass spectrometry (HPLC-MS), which was performed as reported by Dennis and Cheng (2011) and Wang et al. (2023). The cecal content was used to detect the microbiota in feather-pecking ducks and analyze their correlation with neurotransmitters.

### 2.10 Statistical analysis

All statistical analyses were performed using one-way analysis of variance (ANOVA) in SPSS 16.0, and the results are presented as the mean ± SE value. Duncan’s test was used for multiple comparisons. Differences were considered statistically significant at *p* < 0.05. Multiple comparison corrections (False Discovery Rate [FDR]) were not performed. Spearman and Pearson methods were used to conduct a correlation analysis of the relationship between feather-pecking duration and the effects of verbena on microbiota diversity and hypothalamic transmitters. When the *p-*value < 0.05, it represents a significant correlation, and *p* < 0.01 represents an extremely significant correlation.

## 3 Results

### 3.1 Growth performance

Compared with the CON group, no significant differences in weekly body weight were observed in laying ducks across the verbena-based treatments (*p* > 0.05, Table 2). Similarly, the average daily gain (ADG) did not differ significantly among the groups. Although the average daily feed intake (ADFI) in both verbena groups exhibited a decreasing trend, the differences were not statistically significant. The feed conversion ratio (FCR) of the 2% V group (2.47 ± 0.06) and 4% V group (2.42 ± 0.05) was lower than that of the CON group (2.52 ± 0.04) (*p* = 0.014).

**Table 2:** Effects of *verbena officinalis* on the growth performance of growing ducks.

Week	T1(CON)	T2(2%V)	T3(4%V)
Week 1	1200.28 ± 92.06	1224.53 ± 91.36	1204.30 ± 99.47
Week 2	2235.74 ± 96.05	2286.12 ± 86.78	2214.65 ± 106.55
Week 3	3541.22 ± 109.32	3596.57 ± 110.20	3508.52 ± 193.66
Week 4	5043.55 ± 186.79	5076.29 ± 202.33	4928.71 ± 288.58
Week 5	6344.35 ± 268.43	6342.03 ± 246.68	6284.08 ± 255.93
AFDI (g)	419.10 ± 8.72	380.53 ± 12.51	402.52 ± 8.45
ADG (g)	162.55 ± 5 0.78	162.22 ± 6.39	156.13 ± 6.98
FCR	2.52 ± 0.04^a^	2.47 ± 0.06^b^	2.42 ± 0.05^b^

^a,b^ Mean ± SE values with different superscripts in the same row differ (*P* < 0.05); mean ± SE values with the same superscripts or without superscripts in the same row differ (*P* > 0.05).

### 3.2 Serum biochemical parameters

As illustrated in Table 3, no significant differences were observed among the three treatments in serum levels of total protein (TP), albumin (ALB), urea (UREA), or calcium (Ca). However, uric acid (UA) levels in both verbena groups were significantly higher than those in the CON group. On day 42, the serum glucose level in the 4% verbena group was significantly higher than that in the CON group (*p* < 0.01). Although immunoglobulin (Ig) A, IgG, and IgM levels were higher in both verbena groups than in the CON group, these differences were not statistically significant.

**Table 3:** Effects of *verbena officinalis* on serum biochemical indices in growing ducks.

Serum biochemical parameters	T1(CON)	T2(2%V)	T3(4%V)
TP (g/L)	50.44 ± 7.20	50.02 ± 5.87	54.96 ± 9.91
ALB (g/L)	14.76 ± 1.77	14.76 ± 0.94	14.94 ± 1.08
UA (μmol/L)	301.86 ± 26.60^b^	341.72 ± 84.68^a^	340.34 ± 67.64^a^
UREA (mmol/L)	1.65 ± 0.25	1.78 ± 0.35	1.91 ± 0.46
Glucose (mmol)	8.27 ± 2.10^B^	7.67 ± 0.78^B^	11.43 ± 2.16^A^
Ca (mmol)	2.41 ± 0.13	2.47 ± 0.09	2.46 ± 0.08
P (mmol)	1.24 ± 0.42	0.94 ± 0.08 ^b^	1.45 ± 0.48^a^
IgA (g/L)	0.93 ± 0.26	1.07 ± 0.25	1.13 ± 0.24
IgG (g/L)	1.22 ± 0.09	1.31 ± 0.12	1.33 ± 0.18
IgM (g/L)	0.82 ± 0.23	0.85 ± 0.39	0.92 ± 0.25

^a,b^ Mean ± SE values with different lowercase letters as superscripts represent significant differences (*p* < 0.05), whereas distinct uppercase letters indicate highly significant differences (*p* < 0.01).

### 3.3 Cecal microbiota diversity

**Figure 1:**
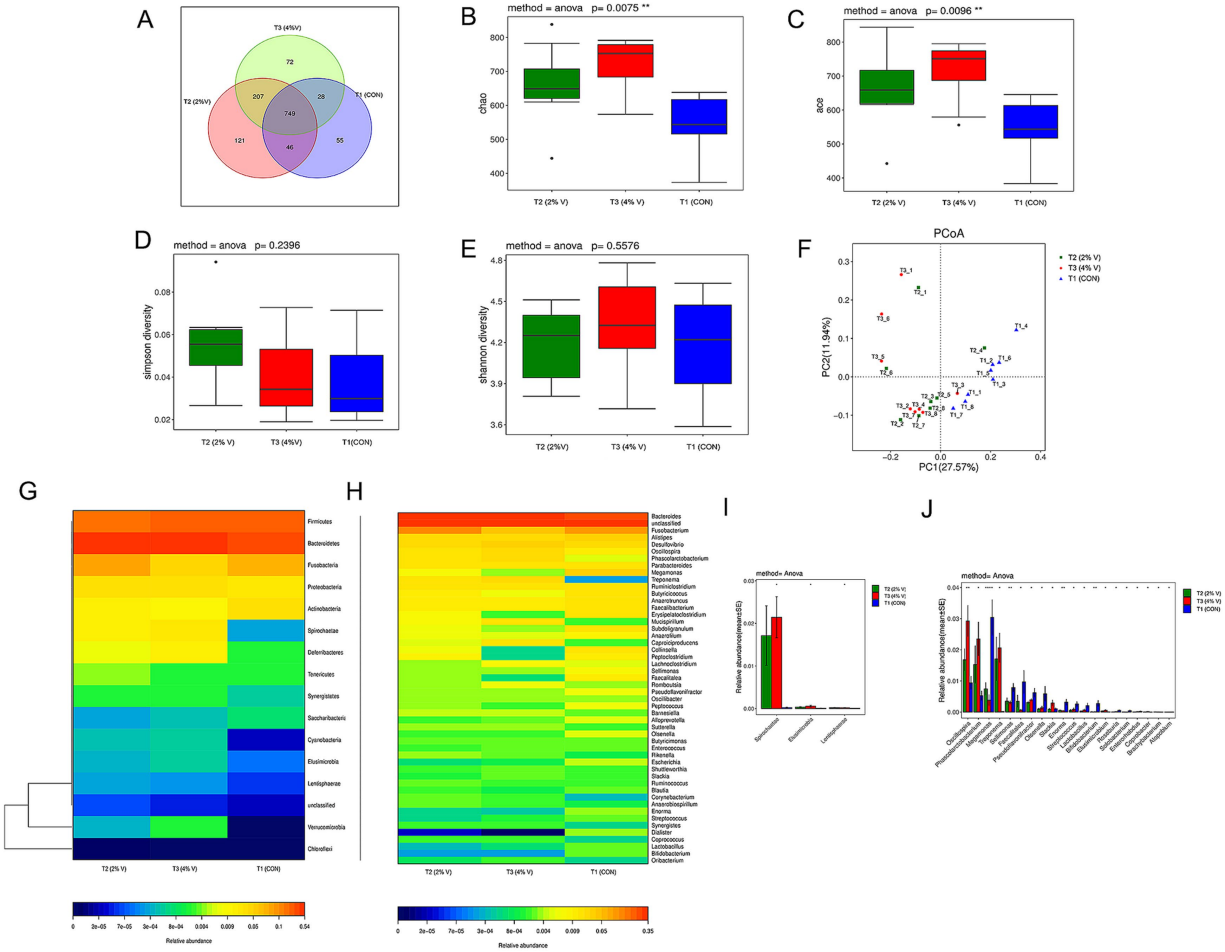
Verbena treatment modulated the species abundance of the cecal microbiota of healthy ducks. **(A)** Venn Diagram of operational taxonomic units (OTUs). **(B–E)** The Chao index (*p* = 0.0075) and ACE index (*p* = 0.0096) of the verbena group were significantly different. The Shannon index (*p* = 0.2396) was slightly higher in the verbena group, while the Simpson index (*p* = 0.5576) was not significantly different. **(F)** PCoA data showed that the *β*-diversity between the 2 and 4% verbena groups was more similar than that between the CON group. **(G)** Heat map of the relative abundance changes in each group at the phylum level. **(H)** The heatmap of relative abundance changes in each group at the genus level. **(I,J)** The histogram showed the relative abundance of different species between the verbena group and the CON group at the phylum and genus levels. T1 (CON) and CON groups. T2 (2% V) and 2% verbena groups. T3 (4% V) and 4% verbena groups.

A Venn diagram was constructed to explore the similarities and differences between the two groups with respect to the microbial communities. The cecal microbial communities of the three groups had 749 common operational taxonomic units (OTUs), with 121 unique OTUs in the 2% verbena group, 72 unique OTUs in the 4% verbena group, and 55 unique OTUs in the CON group (Figure 1A). Supplementation with verbena significantly (*p* < 0.05) increased the number of OTUs.

Alpha diversity (*α*-diversity) of the Chao 1 index of 2%V (659.84 ± 118.47) and 4%V (716.81 ± 89.47) was higher than in the CON group (5543.48 ± 89.40, *p* = 0.007), as well as the ACE index of 2%V (664.91 ± 120.70) and 4%V (714.09 ± 93.47) was higher than in the CON group (545.70 ± 86.35, *p* = 0.009) (Figures 1B,C). The Simpson index was higher in the verbena groups than in the CON group, whereas the Shannon index was lower in the 2% group but higher in the 4% group than in the CON group (*p* > 0.05) (Figures 1D,E). Beta diversity was assessed using principal coordinate analysis (PCoA) to determine the similarity of the gut microbial community structure among the samples. According to the results of the PCoA (Figure 1F), the microbial communities were tightly clustered in the verbena groups but separated from the CON group. The intergroup differences between the groups were higher than the intragroup differences. These results demonstrated that a diet with verbena could significantly alter the flora abundance, diversity, and species differences in the cecal microbiota of ducks.

### 3.4 Difference of cecal microbiota

**Figure 2:**
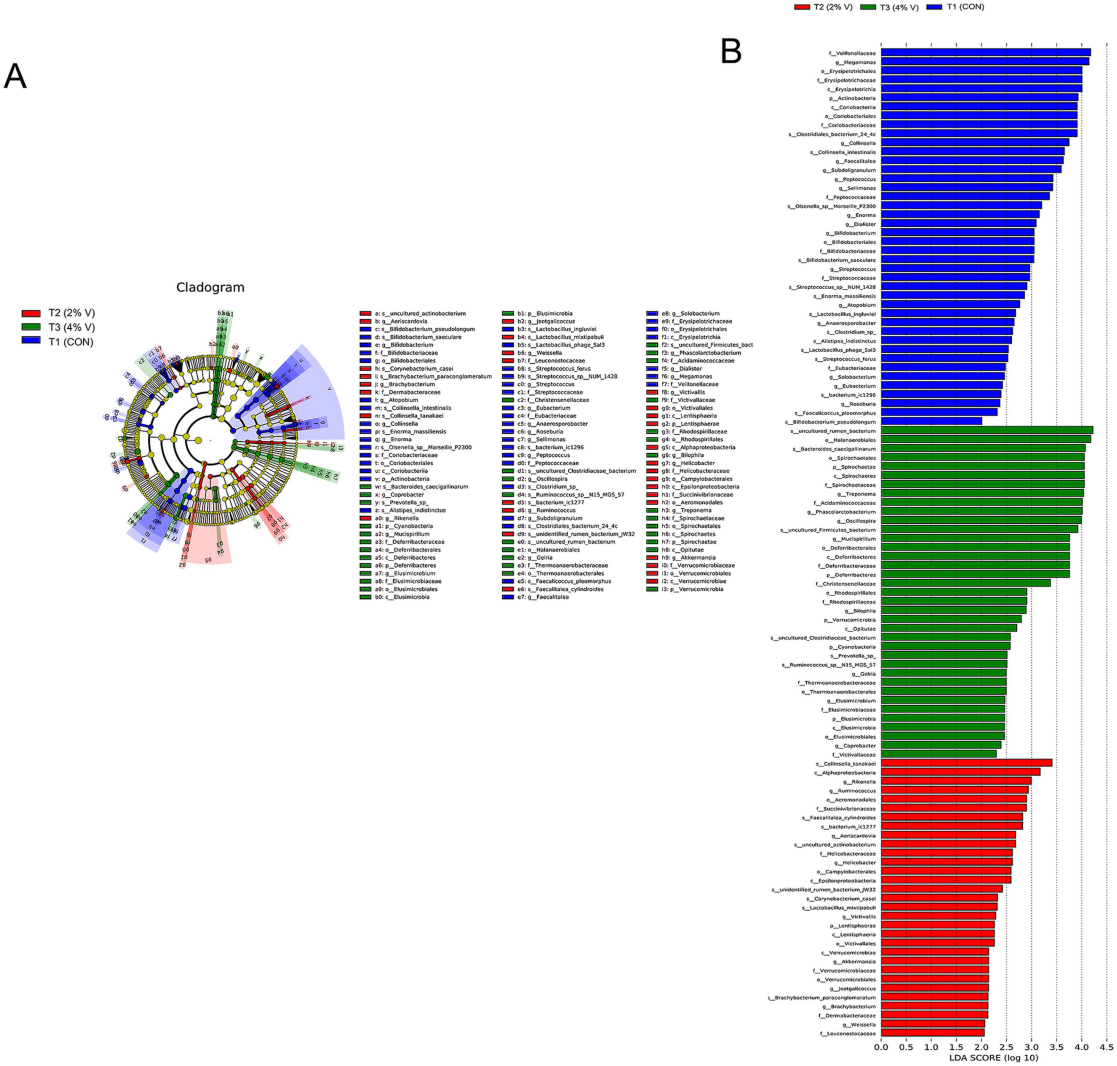
**(A)** Species abundance in the verbena groups and CON groups. **(B)** The LEfSE analysis showed the community composition in the verbena groups and the CON group.

The abundance heatmap of the top species in each group at the phylum and genus levels is displayed in Figures 1G,H. At the phylum level, the abundances of *Bacteroidetes*, *Proteobacteria*, *Spirochaetae*, *Deferribacteria*, *Synergistetes*, *Verrucomicrobia*, *Cyanobacteria*, *Elusimicrobia*, and *Lentisphaerae* were significantly higher in the verbena groups; the abundances of *Firmicutes*, *Actinobacteria,* and *Saccharibacteria* were significantly lower than those in the CON group. At the genus level, compared with the CON group, the abundance of *Bacteroides*, *Desulfovibrio*, *Oscillospira*, and *Treponema* was significantly higher in the verbena groups, whereas *Alistipes* and *Megamonas* were significantly lower in the verbena groups. Additionally, according to the species abundance results, a significant difference analysis was performed at both the phylum and genus levels (Figures 1I,J). *Spirochaetae* was the most divergent phylum, whereas *Oscillospira*, *Phascolarctobacterium*, *Megamonas*, and *Treponema* represented the four most differentially abundant genera. Linear discriminant analysis (LDA) effect size (LEfSe) revealed significant differences in species abundance among the groups. According to LEfSe analysis and LDA score (LDA score ≥ 4), the abundance of *Megamonas* was higher in the CON group than in the verbena group, and *Oscillospira*, *Phascolarctobacterium*, *Treponema*, and *Spirochaetales* were higher in the 4% verbena group (Figures 2A,B). In addition, the MRPP result showed that the A value was 0.009 and the *p-*value was 0.317 at the phylum level, which is consistent with the above results. Collectively, these findings indicate that verbena modulates the composition of the cecal microbiota and enhances the abundance of specific bacterial genera associated with short-chain fatty acid (SCFA) production.

### 3.5 Feather-pecking observations

The durations of the behavioral experiments in feather-pecking ducks are shown in Table 4. Behavior was defined as uninterrupted behavior lasting for more than 4 s aimed at the same bird. We observed 7 days after adding verbena. The results indicated that both verbena supplementation significantly decreased the total duration (*p* = 0.013), and supplementation with 2% verbena significantly decreased the total frequency (*p* = 0.016).

**Table 4:** Duration of feather-pecking behavior after supplementation with *verbena officinalis*.

Items	T1(CON)	T2(2%V)	T3(4%V)
Total frequency (times)	168.22 ± 20.5^a^	121.67 ± 10^b^	165.67 ± 12.1^a^
Total duration (s)	973 ± 6.1^a^	554 ± 15.4^b^	756.21 ± 36.43^b^

^a,b^ Mean ± SE values with different superscripts in the same row differ (*p* < 0.05); mean ± SE values with the same superscripts or without superscripts in the same row differ (*p* > 0.05).

### 3.6 Hypothalamic neurotransmitter

The hypothalamic neurotransmitter levels in feather-pecking ducks are listed in Table 5. Compared to the CON group, the levels of gamma-aminobutyric acid (GABA), histidine (His), tyrosine (Tyr), tryptophan (Trp), and acetylcholine (ACH) in both verbena groups were not significantly different. Serotonin and dopamine were not detected in any sample. Compared to the CON group, glutamine (Gln) levels were lower in the 2% V group (*p* < 0.05) but higher in the 4% V group. Glutamic acid (Glu) levels followed a similar pattern to glutamine (Gln) levels, but the difference was not statistically significant. Noradrenaline (NE) levels in both verbena groups were lower than those in the CON group (*p* < 0.05).

**Table 5:** The concentration of hypothalamic neurotransmitters of feather-pecking ducks.

Indexes	T1(CON)	T2(2%V)	T3(4%V)
GABA (μg/g)	129.60 ± 65.30	131.44 ± 41.71	106.42 ± 46.48
Gln (μg/g)	143.23 ± 9.62	121.35 ± 17.54^B^	171.24 ± 40.74^A^
Glu (μg/g)	326.07 ± 90.64	275.17 ± 91.35	411.55 ± 133.94
His (μg/g)	19.75 ± 7.45	14.54 ± 5.28	12.49 ± 3.99
Tyr (μg/g)	23.67 ± 2.68	20.37 ± 3.67	22.43 ± 8.30
Trp (μg/g)	5.73 ± 0.82	4.33 ± 0.82	4.88 ± 1.56
ACH (μg/g)	0.16 ± 0.007	0.20 ± 0.012	0.21 ± 0.012
NE (μg/g)	0.29 ± 0.014^a^	0.22 ± 0.051^b^	0.25 ± 0.022

^a,b^ Mean ± SE values with different lowercase letters as superscripts represent significant differences (*P* < 0.05), whereas distinct uppercase letters indicate highly significant differences (*p* < 0.01).

### 3.7 The correlation analysis between the cecal microbiota and the hypothalamic neurotransmitter level

**Figure 3:**
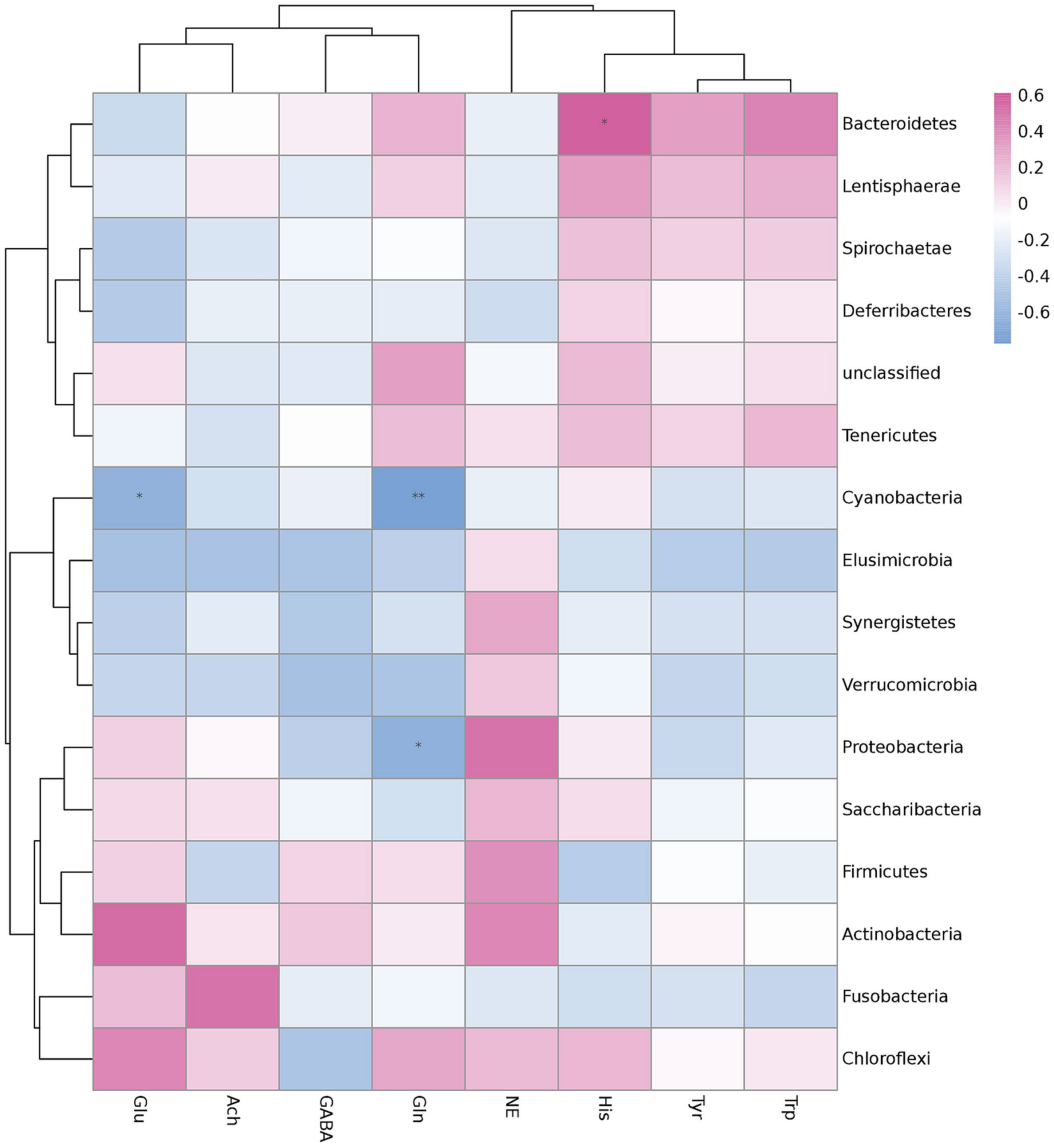
Correlation analysis of hypothalamic neurotransmitters and cecal differential flora in the phylum of feather-pecking ducks. Significant correlations are marked by *p < 0.05 and **p < 0.01.

To determine whether microbiota changes in the cecum were associated with hypothalamic neurotransmitters, Spearman’s correlation analysis was performed. Spearman’s analysis was shown on the heatmap at the phylum level (Figure 3). The results showed that L-glutamine (Gln) was significantly negatively correlated with *Cyanobacteria* (*p* < 0.01) and *Proteobacteria* (*p* < 0.05). L-glutamic acid (Glu) was significantly negatively correlated with *Cyanobacteria* (*p* < 0.05), and L-Histidine (His) was significantly positively correlated with *Bacteroidetes*. Furthermore, noradrenaline (NE) was positively correlated with *Bacteroidetes* and negatively correlated with *Firmicutes*, whereas L-Tryptophan (Trp) showed the opposite result. While the most differential flora, *Spirochaetae*, was positively correlated to His, L-Tyrosine (Tyr), and Trp and negatively correlated to Glu, NE, gamma-aminobutyric acid (GABA), and Acetylcholine chloride (Ach), there was no correlation with Gln.

## 4 Discussion

### 4.1 Effects of verbena on growth performance in healthy laying ducks

*Verbena officinalis* has been extensively studied for its antioxidant, antidepressant, anti-inflammatory, and anticancer effects in animals because of the presence of active ingredients, such as verbascoside and flavonoids (Bahramsoltani et al., 2018). However, the effect of *verbena officinalis* on the growth performance and gut microbiota of ducks has not yet been reported. In the present study, our results demonstrated that dietary *verbena officinalis* did not affect the body weight of growing ducks. These results agree in part with the findings from previous research on the use of verbena or its components in hares (verbascoside concentrations at 1 kg/t and 2 kg/t) (Casamassima et al., 2013), horses (dietary added 0.5 and 1 mg/kg verbascoside) (Palazzo et al., 2019), and even in rainbow trout (dietary added 0.5, 1, and 2% lemon verbena leaf powder) (Hoseinifar et al., 2020). In previous studies by the same author, verbascoside demonstrated a positive effect on the growth rate of suckling lambs (Casamassima et al., 2009) and piglets during the weaning period (Corino et al., 2016). According to Casamassima et al., the observed weight gain is likely associated with the enhancement of milk production induced by herbal supplementation. The ability of *verbena officinalis* to stimulate lactation has been previously reported (Akour et al., 2016). In addition, the duck species in this study was the Guizhou Sansui duck, which is a laying duck species. Sansui ducks exhibit a slow growth rate during the early growth stage. These reasons account for the absence of significant differences in body weight. However, both 2% V and 4% V significantly decreased the feed conversion ratio (FCR). Numerous TCMs and their bioactive components have been proven to be additives that can reduce FCR and promote livestock growth.

### 4.2 Effects of verbena on blood parameters and immune status in healthy laying ducks

Serum biochemical parameters provide a continuous reflection of the health and metabolic dynamics of poultry. Our results showed that different levels of *verbena officinalis* increased the uric acid and glucose. In the polyphenol-enriched compounds, the antioxidant capacity of plasma was associated with an increase in uric acid levels in plasma (Lotito and Frei, 2006). This could explain the increase in uric acid (UA) levels in serum after supplementation with 2 and 4% verbena powder. Studies have demonstrated that uric acid at normal physiological concentrations of UA exhibits antioxidant properties in humans and animals, enhancing endogenous antioxidant capacity, improving immune function, and regulating blood pressure, which may support the antioxidant potential of verbena (Simic and Jovanovic, 1989; Shi et al., 2003; Álvarez-Lario and Macarrón-Vicente, 2010). Serum glucose levels in the 2% V group were lower than those in the 4% V and CON groups.

Glucose is one of the important ingredients in *verbena officinalis* (common verbena) and *Aloysia citrodora*, which are more abundant in the common verbena than in lemon verbena (Polumackanycz et al., 2022). In laying duck production, traditional Chinese medicines, such as fermented or unfermented *Andrographis paniculata* (Liu et al., 2023), rosemary extract (Yao Y. et al., 2023), and silybin (Zhang et al., 2023), have been shown to improve the immune status of laying ducks. Nonetheless, little is known about the effects of *verbena officinalis* on the immunological index of laying ducks. Bekara et al. showed that *verbena officinalis* L. aqueous extract (200 mg/kg) significantly decreased glycemia levels (*p* < 0.05) in adult rats. Conversely, in our study, glucose levels were higher in the 4% verbena group than in the CON group (*p* < 0.01). Thus, the effect of verbena on serum glucose levels requires further study. Relative to the control group, 2% V and 4% V demonstrated a potential to elevate the concentrations of immunoglobulins IgA, IgG, and IgM, despite the lack of significant differences. These findings align with the study by Pastorelli et al., who reported that dietary supplementation with *verbena officinalis* extract significantly increased serum IgA concentrations in piglets (Pastorelli et al., 2012).

### 4.3 Effects of verbena on cecal microbiota in healthy laying ducks

The gut microbiota and its metabolites play an important role in duck growth performance and disease prevention (He et al., 2019; Lyu et al., 2021). Silva et al. reported that, under simulated gut conditions, a complex composed of hibiscus and lemon verbena (brand name Metabolaid®), in a dose-dependent manner, modulated the gut bacteriome to higher relative abundances of some major genera (>3% relative abundance), such as *Bifidobacterium*, *Blautia,* and *Faecalibacterium*, leading to an increase in the relative abundance of *Prevotella* and *Akkermansia* (Silva et al., 2022), which indicated that Metabolaid® might exert beneficial gut microbiome-modulating properties *in vivo*. Diez-Echave et al. (2020) reported that *Lippia citriodora* extract reduces intestinal dysbiosis by reducing the *Firmicutes*/*Bacteroidetes* ratio and increasing *Akkermansia* abundance in comparison with untreated HFD mice. These results suggest that verbena has some positive effects on gut health.

In the present study, both verbena groups increased the relative abundance of *Bacteroidetes* and decreased the relative abundance of *Firmicutes*, which indicated the *Firmicutes*/*Bacteroidetes* ratio is reduced. A recent study reported that supplementation with *Bacillus toyonensis* BCT-7112^T^ in ducks increased microbial diversity and shifts in cecal microbiota populations, particularly enhanced levels of *Bacteroidetes* and decreased pathogenic genera such as *Fusobacterium* (Incharoen et al., 2025). Our results showed comparable shifts toward beneficial microbiota with dietary *verbena officinalis*. Furthermore, Incharoen et al. demonstrated that gut microbiota modulation positively influences production performance (e.g., egg quality and ammonia emissions) through dietary supplements. For the differential microbiota in our results, the relative abundance of *Spirochaeta*, *Elusimicrobi*, and *Lentisphaera* was higher (*p* < 0.05) in the verbena groups compared with the CON group. *Oscillospira* and *Phascolarctobacterium* were the two most significantly enriched genera (*p* < 0.01, *p* < 0.05) in the verbena group, whereas *Megamonas* and *Sellimona* were the two most abundant (*p* < 0.01) genera in the CON group. However, the effects of different TCMs, probiotics, or plant extracts on the gut microbiota of ducks are different. *Echinacea* extract (EE) increased the relative abundance of *Bacteroides* and significantly decreased the relative abundance of *Megamonas* in immunosuppressed ducks at 28 days of age compared with controls (Yang et al., 2021; Lin et al., 2022). Similar results were obtained.

When verbena is added to diets, there is no research reporting an increase in *Oscillospira* and *Phascolarctobacterium* in the cecum. Notably, *Oscillospira* has a strong association with health. This genus possesses the ability to synthesize short-chain fatty acids (SCFAs), including butyrate, which are acknowledged as “next-generation probiotics” (Yang et al., 2021). SCFAs improve poultry intestinal health by enhancing energy metabolism, maintaining mucosal integrity, modulating immune homeostasis, and optimizing the microbial composition (Yang H. et al., 2020; Ali et al., 2022). Furthermore, *Phascolarctobacterium* belongs to the *Verrucaceae* family, which is part of the *Firmicutes* phylum and produces SCFAs (Bucher-Johannessen et al., 2024), including acetate and propionate. These compounds might be associated with the metabolic state and mood of the host. These results mirrored those associated with hibiscus (*Hibiscus sabdariffa*) and lemon verbena (*Lippia citriodora*), exemplified by the commercial product Metabolaid®, which has demonstrated the ability to boost SCFAs production (Silva et al., 2022). Another study conducted on Gilthead Seabream (*Sparus aurata*) revealed that a combination of transcriptomics and histological findings indicated that extracts from the medicinal plant leaf extract (MPLE) from sage (*Salvia officinalis*) and lemon verbena (*Lippia citriodora*) could sustain intestinal health and enhance the integrity of the intestinal epithelium. This suggests that this extract may be a promising additive for aquafeeds (Salomón et al., 2021).

Notably, both verbena groups decreased the relative abundance of *Lactobacillus* and *Bifidobacterium* in the present study. However, gut microbial catabolism of polyphenols is characterized by large individual variability (Morand and Tomás-Barberán, 2019). Another study reported that the extracts of hibiscus and lemon verbena exhibited a dose-dependent increase in the relative abundances of *Bifidobacterium*, *Blautia*, *Faecalibacterium*, *Prevotella*, and *Akkermansia* in human fermented fecal samples (Lyu et al., 2021).

In traditional medicine, verbena has been demonstrated to possess therapeutic potential for neurological conditions (Kubica et al., 2020). However, previous research has primarily focused on behavioral studies in mice (Lai et al., 2006; Khan et al., 2016; Rashidian et al., 2017). To date, no studies have demonstrated the behavioral effects of verbena on ducks under controlled experimental conditions. Therefore, we believe that the behavioral effect of verbena on ducks is promising but still requires mechanistic studies.

### 4.4 Effects of verbena on feather-pecking behavior by altering cecal microflora composition and hypothalamic neurotransmitter levels

Research on feather-pecking behavior has demonstrated that dietary interventions, such as the inclusion of nutrients, trace elements, and neuroactive compounds, can effectively improve gut microbiota composition, neurotransmitter levels, and metabolic functions, ultimately mitigating feather-pecking behavior (Kjaer and Bessei, 2013). Although the beneficial effects of verbena on growth performance, immune function, and antioxidant capacity have been reported in species such as pigs, chickens, horses, and aquatic animals, its influence on feather-pecking behavior in laying ducks remains unexplored. In this study, dietary supplementation with verbena changed the cecal microbiota composition and significantly decreased pecking duration. Mindus et al. reported that oral *L. rhamnosus* supplement (Lacto group) prevented the stress-induced increase of severe FP behavior and improved feather cover, but the levels of TRP, PHE, and TYR were not changed (Kraimi et al., 2019). Our results are similar to this finding. Therefore, we consider that adding verbena to the diet is of great significance to reduce the pecking behavior of ducks.

According to recent findings, pecking behavior in birds correlates with neurotransmitter imbalances, similar to those observed in human psychiatric disorders (Widner et al., 2002; Birkl et al., 2019). Tryptophan (Trp) and noradrenaline (NE) are important neurotransmitters in the study of feather-pecking behavior (Kops et al., 2017). In this study, compared to the CON group, the levels of gamma-aminobutyric acid (GABA), histidine (His), tyrosine (Tyr), tryptophan (Trp), and acetylcholine (ACH) in both verbena groups were not significantly different. Serotonin and dopamine were detected in these samples. The glutamine (Gln) level was lower in the 2% V group (*p* < 0.05) but higher in the 4% V group. Glutamic acid (Glu) levels followed a similar pattern to glutamine (Gln) levels, but the difference was not statistically significant. Noradrenaline (NE) levels in both verbena groups were lower than the CON group (*p* < 0.05). Noradrenaline is a catecholamine that is, in the majority of cases, associated with the “fight-or-flight” response (Haden and Scarpa, 2007). Studies on noradrenergic activity and aggressive behavior have shown that noradrenergic levels increase when humans and animals act aggressively (Dennis and Cheng, 2011; Dennis, 2016). Korte et al. (1997) demonstrated that HFP (high feather pecking) hens respond to restraint with significantly greater plasma NE than LFP (low feather pecking) hens (Korte et al., 1997). There are some controversies about the relationship between NE levels and aggressive behavior in poultry, but NE levels generally increase immediately when birds have aggressive behavior (Dennis and Cheng, 2012; Dennis, 2016; Mindus et al., 2021). Our results suggest that the reduction in feather-pecking behavior in laying ducks supplemented with *verbena officinalis* may be associated with decreased norepinephrine (NE) levels in the hypothalamus.

Meanwhile, 2 and 4% verbena diets increased the relative abundance of *Spirochaetae*, which was negatively correlated with the NE level. Simultaneously, both verbena groups experienced an increase in the abundance of *Bacteroidetes*, which had a negative correlation with NE; the correlation between *Firmicutes* and NE showed the opposite result. These results suggest that verbena could reduce feather-pecking behavior by decreasing the NE level, accompanied by a decrease in the abundance of *Firmicutes* and an increase in the relative abundance of *Bacteroidetes* and *Spirochaetes* at the phylum level.

A recent study reported that the hippocampal glutamate levels were significantly increased in FP chickens (Yan et al., 2025). In the present study, the glutamic acid (Glu) level in the 2% V group was lower than the CON group. The correlation analysis indicated that the hypothalamic glutamate levels were significantly negatively correlated with *Cyanobacteria* (*p* < 0.05). The second experimental stage revealed that supplementation with *verbena officinalis* led to an increasing trend in *Cyanobacteria*, although the change was not statistically significant. These results may suggest that a diet supplemented with *verbena officinalis* can reduce the feather-pecking frequency and duration by increasing the abundance of *Cyanobacteria,* accompanied by a decrease in hypothalamic glutamate. However, the regulatory pathways involved require further study.

## 5 Conclusion

In conclusion, our findings indicate that dietary supplementation with *verbena officinalis* powder enhances partial immune parameters and alters the composition of cecal microbiota in healthy ducks. Both *verbena officinalis* levels increased the relative abundance of *Bacteroidetes* and decreased the relative abundance of *Firmicutes* at the phylum level and *Megamonas* at the genus level. Following dietary supplementation of *verbena officinalis* in feather-pecking ducks, we observed a significant reduction in the duration of feather-pecking behavior. Correlation analysis revealed that this reduction in feather-pecking behavior may be associated with changes in hypothalamic norepinephrine (NE) levels. Based on the evaluation of growth performance, serum biochemical parameters, cecal microbiota changes, feather-pecking duration reduction, hypothalamic neurotransmitter changes, and economic considerations, we recommend the inclusion of 2% *verbena officinalis* in the diet of ducks. This supplementation not only reduces the feed conversion ratio (FCR) and promotes intestinal development but also alleviates aggressive feather-pecking behavior. However, the current study did not include morphological observations of the gut, and the relationship between feather-pecking behavior and the gut-brain axis requires further exploration. These aspects should be addressed in future studies.

## Data Availability

The data presented in the study are deposited in the National Center for Biotechnology Information (NCBI) under the BioProject number PRJNA1269526 (http://www.ncbi.nlm.nih.gov/bioproject/1269526). All other data are available upon request to the corresponding author.

## Glossary

CON: control
FP: feather pecking
TCM: traditional Chinese medicine
ADFI: average daily feed intake
ADG: average daily gain
FCR: feed conversion ratio
ALB: albumin
TP: total protein
UA: uric acid
UREA: urea
P: phosphorus
Ca: calcium
IgA: immunoglobulin A
IgG: immunoglobulin G
IgM: immunoglobulin M
GABA: gamma-aminobutyric acid
His: histidine
Tyr: tyrosine
Trp: tryptophan
Gln: glutamine
Glu: glutamic acid
NE: noradrenaline
SCFAs: short-chain fatty acids
